# Postpartal Affective and Endocrine Differences Between Parents of Preterm and Full-Term Infants

**DOI:** 10.3389/fpsyt.2020.00251

**Published:** 2020-04-01

**Authors:** Tobias Weigl, Nora Schneider, Anja Stein, Ursula Felderhoff-Müser, Manfred Schedlowski, Harald Engler

**Affiliations:** ^1^Institute of Medical Psychology and Behavioral Immunobiology, University Hospital Essen, University of Duisburg-Essen, Essen, Germany; ^2^Psychology School, Fresenius University of Applied Sciences Düsseldorf, Düsseldorf, Germany; ^3^Department of Pediatrics I, Neonatology, University Hospital Essen, University of Duisburg-Essen, Essen, Germany

**Keywords:** preterm birth, postpartum, depression, anxiety, cortisol, ovarian hormones

## Abstract

**Background:**

During the postpartum period, new parents frequently experience emotional stress and exhibit symptoms of depression and anxiety, accompanied by substantial endocrine changes. However, evidence predominantly exists from parents of full-term infants, while data on parents of preterm infants are scarce. In this exploratory, cross-sectional study, we compared psychological well-being and endocrine parameters in parents of very preterm and term born infants.

**Methods:**

Mothers (N = 28) and fathers (N = 30) of full-term infants as well as mothers (N = 18) and fathers (N = 21) of very or extreme preterm infants (< 32^nd^ gestational week) were recruited in the days following birth. Anxiety, depression, and perceived stress were assessed with the State-Trait Anxiety Inventory (STAI), the Beck Depression Inventory (BDI), and the Perceived Stress Questionnaire (PSQ), respectively. Physiological measures included serum levels of estradiol, progesterone, prolactin, and thyroid-stimulating hormone (mothers only), as well as the salivary cortisol awakening response (mothers and fathers).

**Results:**

New mothers and fathers of very preterm infants exhibited higher scores of depression, anxiety and stress than parents of term infants. Besides, mothers of very preterm infants showed lower levels of estradiol, progesterone, and prolactin, as well as a heightened post-awakening cortisol response compared to mothers of term infants. Furthermore, in mothers of preterm infants we found significant negative associations between serum prolactin levels and BDI and STAI scores, respectively.

**Conclusions:**

Parents of very preterm infants suffered from a higher burden of psychological distress than parents of full-term infants. The affective symptoms in preterm mothers were accompanied by altered endocrine profiles that, at least to some extent, may contribute to the psychological changes. The profound psychological and physiological disturbances in mothers of preterm infants may have an impact on long-term mental health and early pharmacological and psychological interventions may help to ameliorate postpartum affective symptoms.

## Introduction

Becoming a parent is usually an occasion of great joy and most parents rate having a baby among their most significant life events. However, women may experience this event as quite painful, in some cases even traumatic, e.g., due to medical complications affecting either child, mother, or both (1). Besides, recent parenthood requires enormous efforts to adapt to considerable changes in life circumstances, with a shift in social roles, alterations in daily routine including sleep deprivation, regular nursing or feeding, and taking care of the newborn (2). Due to these challenges new mothers and fathers are more vulnerable to develop symptoms of psychological distress compared to the general population. Certainly, the most prominent psychological disorder in mothers is postpartum depression (PPD), affecting up to 20% of mothers within 3 months after delivery (3). Moreover, up to 21% of mothers suffer from anxiety in the days following birth (4).

During pregnancy and puerperium, the tremendous endocrine changes in the expecting or new mothers might play a critical role in the development of depression, anxiety, or the experience of stress (5). For example, concentrations of estradiol and progesterone continuously rise during pregnancy and rapidly decline after birth. The pronounced postpartum drop in these hormones to a hypogonadal state is thought to trigger the development of depressive symptoms, as stated in the withdrawal-theory of PPD (6, 7), and estradiol therapy has been shown to ameliorate PPD (8, 9). For the pituitary hormone prolactin, which exhibits anxiolytic and stress-relieving properties (2, 10), a negative association between serum levels and anxiety scores in nursing mothers has been found (11). However, the role of prolactin in PPD remains unclear (12). Furthermore, the thyroid-stimulating hormone (TSH) has been proven to be involved in the development of depression, yet its role for PPD still remains elusive (13). While TSH within normal levels is not associated with PPD in general, women with levels above the standard range show a higher probability to develop symptoms of PPD 6 months after delivery. Nevertheless, the thyroid function 48 h after delivery did not predict symptoms of PPD (14, 15). Furthermore, symptoms of depression, anxiety, and stress may also be connected to altered cortisol secretion. In this context, it has been shown that cognitive-behavioral treatment before birth significantly attenuated the cortisol awakening response (CAR) 2 months following delivery. However, this result was only seen in mothers with elevated symptoms of psychological distress before birth (16).

Even though men are not affected by physiological or endocrine changes related to pregnancy, the postpartal period also seems to be a time of increased vulnerability for fathers as reflected by a heightened depression rate compared to the general population (17). Although certain works even presume a similar rate of PPD in women and men (18, 19), only few studies focused on depression in men which probably accounts for an underestimation of depression in new fathers compared to mothers (20). Data on postpartum anxiety in fathers are sparse as well. However, in a sample of 260 fathers 8.5% exhibited high anxiety measured in the first three days after birth (21). Assumedly, parents of babies born preterm even suffer from higher psychological distress than parents of babies born at full term, reflected by more pronounced levels of depression and anxiety in mothers (21–25). Even 1 year after birth of a very preterm baby, parenting stress in mothers was double when compared to mothers of full-term babies (26).

Despite emerging evidence indicating that birth can have detrimental consequences on psychological health in parents, data on the effects of preterm birth remain scarce. Moreover, to our knowledge, only few studies on fathers exist so far (17, 25). Yet most fundamental, studies on the etiology of postpartum mental disorders lack integration of biological, psychological, and psychosocial measures (27). This is complicated by the fact that antenatal depression, anxiety, and stress burden seem to be independent risk factors for preterm birth (28). Regarding endocrine measures, there is evidence that a decline of progesterone may lead to preterm birth. However, no causal relationship with preterm birth could be established so far (29, 30). Even though the direction of causality remains elusive, it is highly important to further identify relevant psychological and physiological factors that are associated with preterm birth. Therefore, this explorative study aimed at investigating differences in psychological and physiological characteristics in parents of preterm infants compared to parents of term infants, assuming a higher psychological burden in parents of preterm infants. For this purpose, we assessed levels of depression, anxiety, and perceived stress as well as blood and saliva concentrations of different endocrine factors within 6 days after birth. Additionally, we explored whether psychological symptoms were related to endocrine measures.

## Material and Methods

### Study Population

New parents of very or extreme preterm infants admitted to the NICU (< 32 weeks of gestation; “Preterm” group) and full-term infants (≥37 weeks of gestation; “Term” group), were recruited between September 2010 and September 2013 in the Department of Pediatrics I/Neonatology and the Department of Gynecology and Obstetrics at the University Hospital Essen, Germany. All eligible parents received both oral and written information about the study objectives and were told about the possibility to withdraw from the study any time. After written informed consent was obtained, participants were offered to make use of mental health support (i.e., psychological psychotherapists and/or psychiatrists) if needed. The study protocol has been approved by the Ethics Review Board of the University of Duisburg-Essen (approval no. 09-4239) and was performed in accordance with the Declaration of Helsinki. Initially, a total of 101 mothers and 92 fathers gave written informed consent to follow the study protocol as briefed by study personnel. However, for various reasons, 55 mothers (Term: N = 36, Preterm: N = 19) and 41 fathers (Term: N = 29, Preterm: N = 12) did not complete the study. Finally, questionnaires and blood and saliva samples of a total of 46 nursing mothers (Term: N = 28, Preterm: N = 18) and 51 fathers (Term: N = 30, Preterm: N = 21) could be obtained. In both groups, parents were excluded for multiple births, death of the child during the study, younger age than 18 years of at least one parent, insufficient German language skills, previously diagnosed psychiatric disorders (e.g., depression, alcohol abuse), serious medical states (e.g., pre-eclampsia). Specific exclusion criteria for parents of term infants were medical conditions of infants which required inpatient treatment, instrumental vaginal birth (i.e., use of forceps or ventouse), and emergency C-section. To exclude lactation-related differences in endocrine measures (i.e., estradiol, progesterone, prolactin, TSH, and cortisol), only breastfeeding mothers and mothers using breast pumps were included in the study.

### Study Design

Recent parents of preterm and term infants were invited on the second day after birth to participate in a study on parental psychological and physiological well-being in the postpartum period. Cross-sectional data included psychological and endocrine measures and were all obtained within 6 days after birth. Participants filled in a set of questionnaires, consisting of a self-administered questionnaire regarding obstetric and demographic information, and questionnaires assessing depression, anxiety, and perceived stress. From mothers only, a blood sample was collected to assess estradiol, progesterone, prolactin, and TSH concentrations. Additionally, saliva for the assessment of different measures of the CAR was collected by all mothers and fathers on two consecutive days.

### Psychological Questionnaires

#### Beck Depression Inventory

Depressive symptoms were assessed with the Beck Depression Inventory (BDI), a well-established self-report measure consisting of 21 items to assess cognitive, affective, and somatic symptoms of depression in the week prior to assessment. Each item offers a choice of four ordinal statements which reflect an increasing range of severity, ranging from 0 to 3, with a total possible score range from 0 to 63. Scores above 18 indicate clinically significant depression, whereas scores from 11 to 17 mark mild to moderate depression. Non-depressed persons or patients in remission typically exhibit scores <11 (31).

#### State-Trait Anxiety Inventory

State anxiety was assessed with the 20-item state version of the State-Trait Anxiety Inventory (STAI-S) (32). Each of the Likert-scaled items offers the rating on a four-point scale from “almost never” (1) to “almost always” (4). Possible total scores range from 20 to 80 with higher scores reflecting higher state anxiety.

#### Perceived Stress Questionnaire

The level of perceived stress was quantified using the 20-item Perceived Stress Questionnaire (PSQ) (33). Each item provides a rating on a four-point scale, indicating how often the individual is concerned by the item, ranging from “almost never” (1) to usually (4). An overall score is computed taking all items into account, with higher sum scores indicating more severe perceived stress.

### Endocrine Analyses

Blood for endocrine analyses was collected in tubes containing clot activator (S-Monovette, Sarstedt, Nuembrecht, Germany). Samples were immediately transferred to the Central Diagnostic Laboratory at the University Hospital Essen and serum concentrations of estradiol, progesterone, prolactin, and TSH were measured using electrochemiluminescence immunoassays. For estradiol, the Roche Modular analytics E170 module (Roche Diagnostics, Mannheim, Germany) and for progesterone, prolactin and TSH, the Siemens Advia Centaur system (Siemens Healthcare Diagnostics, Eschborn, Germany) were used. The concentration of cortisol in saliva was determined using a commercial enzyme-linked immunosorbent assay (Cortisol ELISA, IBL International, Hamburg, Germany) according to the manufacturer’s instructions. Cross-reactivity of the anti-cortisol antibody with other relevant steroids was 7.0% (11-deoxycortisol), 4.2% (cortisone), 1.4% (corticosterone), 0.35% (progesterone), and <0.01% (testosterone, estrone, estradiol, estriol). Intra- and interassay variances were 4.8% and 5.9%, respectively. The detection limit was 0.138 nmol/l.

### Cortisol Awakening Response

For assessment of the CAR, participants were asked to collect saliva samples on two consecutive mornings at the following time points: immediately upon awakening, as well as 30, 45, and 60 min after awakening. Saliva was collected with a commercial collection device (Salivette^®^ Cortisol; Sarstedt, Nuembrecht, Germany). To ensure accurate saliva sampling, participants were instructed in detail about the procedure by trained study personnel, including the practice of relevant aspects. Additionally, participants received written take-home instructions for use at home with the sampling devices. The aforementioned procedures were used to maximize participant adherence, which is in line with recent consensus guidelines (34). Subjects were asked not to eat, drink (except water), smoke or brush their teeth before the end of the sampling to avoid any contamination. Apart from the aforementioned restrictions, participants were allowed to follow their everyday routine. Compliance to protocol was assessed by a self-report questionnaire. Long-term stability of salivary cortisol allows returning of samples up to 2 weeks after collection (35). Saliva samples were sent back by mail to the laboratory, where saliva was obtained by centrifugation and stored at −80° until analysis. We calculated the area under the curve with respect to ground (AUCg) and with respect to increase (AUCi) according to Pruessner et al. (36). The AUCg provides an estimate of the cortisol secretion in total over the first hour after awakening. To provide a measure of the dynamics (changes) of the CAR, the AUCi was used.

### Statistical Analysis

Statistical analyses were performed using PASW statistics version 25 for windows (SPSS, Chicago, IL, USA). Several variables violated the normality assumption in *a priori* testing for deviations from normal distribution with skewness, kurtosis, and the Kolmogorov–Smirnov test. Thus, statistical analyses were based on non-parametric testing to provide an effectual robust method (37). Differences between groups in sociodemographic, psychological, and physiological variables were analyzed employing Mann–Whitney U tests and Chi-square (X^2^) test (for dichotomous variables). Correlations were calculated using Spearmans rho. Results are displayed as mean ± standard error of the mean (SEM).

## Results

### Sample Characteristics

Mothers and fathers of infants born at term or preterm did not differ in age as well as birth characteristics like parity or sex of infant. Infants’ birth weight and length of gestation were significantly lower in the Preterm group. While relationship status did not differ between groups, parents of term infants were higher educated and holding a university degree more often than parents of preterm infants (see **Tables 1** and **2** for further details).

**Table 1:** Sample and group characteristics of mothers and corresponding infants.

Variables	Term (n = 28)	Preterm (n = 18)	*p*-Value
Age of mothers (years)^1^	29.5 ± 4.3 (21–43)	30.5 ± 6.2 (18–43)	0.470^a^
Body mass index of mothers^1^	24.9 ± 4.6 (18–36)	24.7 ± 3.5 (19–32)	0.928^a^
Length of gestation (weeks)^1^	39.3 ± 0.9 (38–41)	28.3 ± 2.3 (23–31)	< 0.001^a^
Infant birth weight (gram)^1^	3320.5 ± 436.9 (2670–4600)	1198.0 ± 365.0 (450–1810)	< 0.001^a^
Parity (N/%)			0.097^b^
Primiparous	15/53.6	14/77.8	
Multiparous	13/46.4	4/22.2	
Infant sex (N/%)			0.683^b^
Female	17/60.7	12/66.7	
Male	11/39.3	6/33.3	
Relationship status (N/%)			0.091^b^
Married	22/78.6	9/50.0	
Cohabitation	6/21.4	8/44.4	
Divorced/living apart	–	1/5.6	
Education (N/%)			0.017^b^
No degree	–	1/5.6	
Secondary education	7/25	8/44.4	
Degree to enter university	19/67.9	4/22.2	
Other	2/7.1	5/27.8	
Profession (N/%)			0.025^b^
No training	1/3.6	4/22.2	
Still in vocational training	4/14.3	–	
Completed vocational training	6/21.4	8/44.4	
Degree from professional school	1/3.6	2/11.1	
University degree	15/53.6	3/16.7	
Other	1/3.6	1/5.6	

^1^M ± SD/(range).

a Mann–Whitney.

b Chi-square test.

**Table 2:** Sample and group characteristics of fathers and corresponding infants.

Variables	Term (n = 30)	Preterm (n = 21)	*p*-Value
Age of fathers (years)^1^	32.6 ± 5.7 (20–47)	33.2 ± 7.6 (18–46)	0.667^a^
Body mass index of fathers^1^	25.6 ± 2.9 (19–32)	27.7 ± 4.6 (21–39)	0.268^a^
Length of gestation (weeks)^1^	39.3 ± 1.1 (37–42)	28.5 ± 2.0 (25–32)	< 0.001^a^
Infant birth weight (gram)^1^	3275.7 ± 411.6 (2590–4600)	1248.8 ± 382.9 (460–1810)	< 0.001^a^
Parity (N/%)			0.838^b^
Primiparous	18/60.0	12/57.1	
Multiparous	12/40.0	9/42.9	
Infant sex (N/%)			0.382^b^
Female	18/60.0	10/47.6	
Male	12/40.0	11/52.4	
Relationship status (N/%)			0.261^b^
Married	22/73.3	12/57.1	
Cohabitation	7/23.3	9/42.9	
Divorced/living apart	1/3.3	–	
Education (N/%)			0.002^b^
No degree	–	3/14.3	
Secondary education	8/26.7	10/47.6	
Degree to enter university	21/70.0	6/28.6	
Other	1/3.3	2/9.5	
Profession (N/%)			0.197^b^
No training	3/10.0	6/28.6	
Still in vocational training	2/6.7	1/4.7	
Completed vocational training	5/16.6	6/28.6	
Degree from professional school	2/6.7	2/9.5	
University degree	18/60.0	6/28.6	
Other	–	–	

^1^ M ± SD/(range).

a Mann–Whitney.

b Chi-square test.

### Group Comparisons Between Mothers

#### Depression, Anxiety, and Stress Scores in Mothers

**Figure 1:**
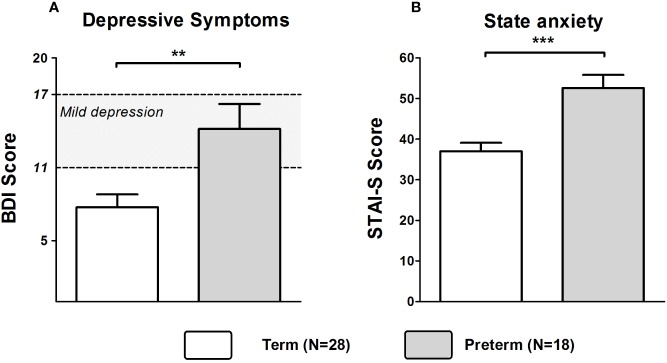
Scores of BDI **(A)** and STAI-S **(B)** in mothers of infants born at term or preterm. ***p < 0.001; **p < 0.01. Data are shown as mean values ± SEM.

Mothers of the Preterm group showed significantly higher depression scores as assessed by the BDI (Z = −2.818, *p* = 0.005). Based on published cutoffs, the mean BDI score of mothers in the Preterm group reflected a state of mild to moderate depression reached by about 55% of mothers in this group (see **Figure 1A**). State anxiety was also significantly higher in the Preterm compared to Term group (Z = −3.638, *p* < 0.001; see **Figure 1B**). Additionally, levels of perceived stress were significantly higher in mothers of the Preterm group compared to the Term group (Z = −2.242, *p* = 0.025).

#### Estradiol, Progesterone, Prolactin, and TSH in Mothers

**Figure 2:**
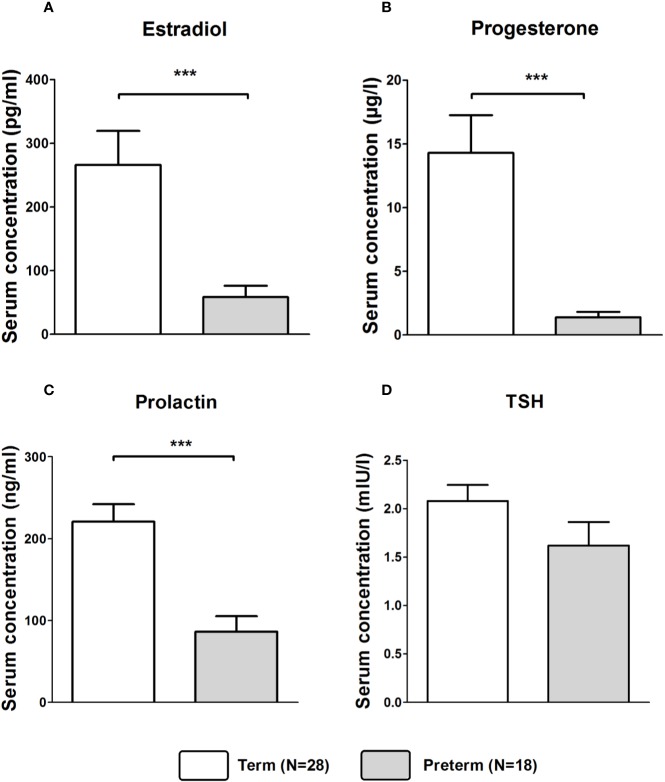
Serum concentrations of estradiol **(A)**, progesterone **(B)**, prolactin **(C)**, and TSH **(D)** in in mothers of infants born at term or preterm. ***p < 0.001. Data are shown as mean values ± SEM.

Mann–Whitney U tests revealed that serum concentrations of estradiol (Z = −4.209, *p* < 0.001; **Figure 2A**), progesterone (Z = −5.044, *p* < 0.001; **Figure 2B**), and prolactin (Z = −4.344, *p* < 0.001; **Figure 2C**) were significantly lower in the Preterm compared to the Term group. Serum concentrations of TSH did not differ significantly between groups (**Figure 2D**).

#### Post-Awakening Cortisol Response in Mothers

**Figure 3:**
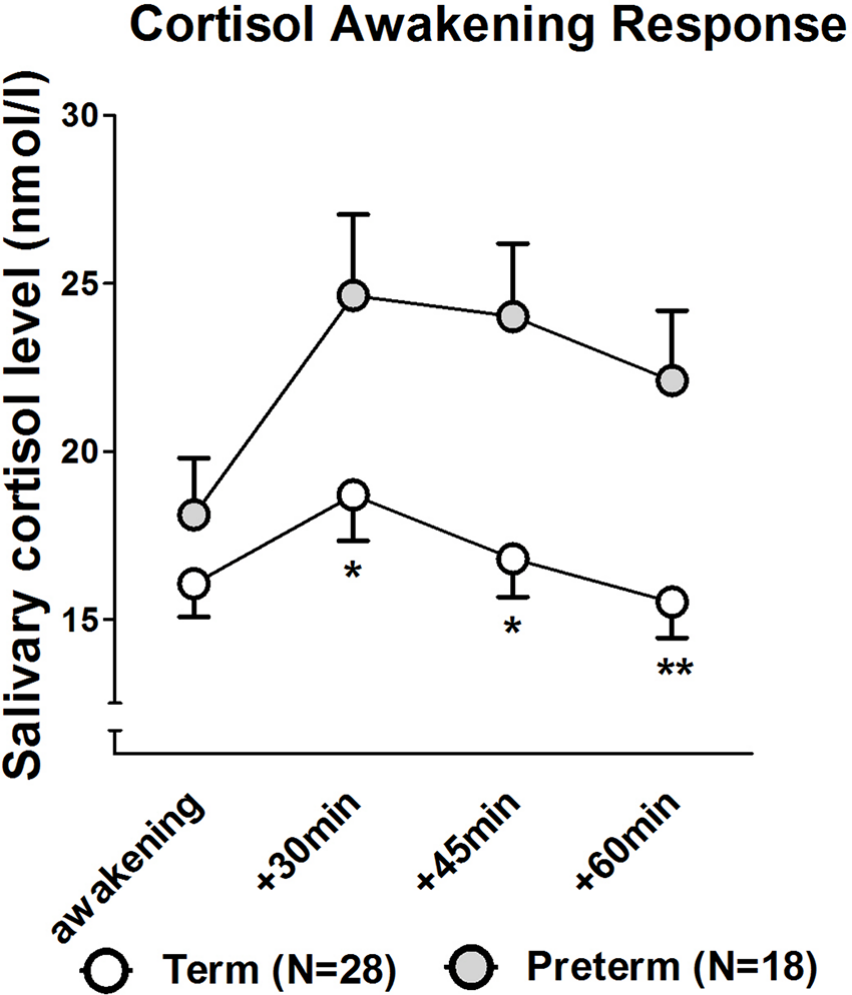
Cortisol awakening response in mothers of infants born at term or preterm. **p < 0.01; *p < 0.05. Data are shown as mean values ± SEM.

Salivary cortisol concentration at awakening did not differ significantly between Preterm and Term groups. However, Preterm mothers exhibited a significantly higher CAR as evident from higher salivary cortisol levels at 30, 45, and 60 min post-awakening (all *p* < 0.05; see **Figure 3** for further details) as well as significant group differences in AUCg (Z = −2.453, *p* = 0.014) and AUCi (Z = −2.026, *p* = 0.043).

#### Associations Between Endocrine and Psychological Parameters in Mothers

**Figure 4:**
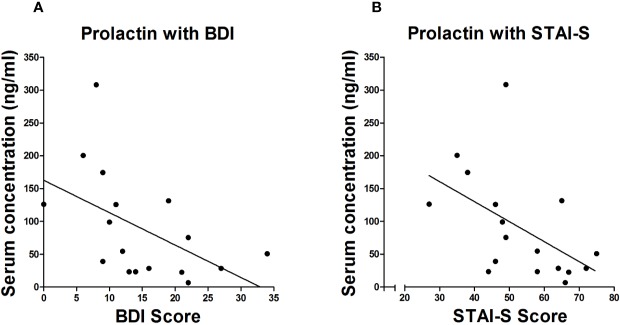
Spearman correlation between serum levels of prolactin and scores of BDI **(A)** and STAI-S **(B)** in mothers of infants born preterm.

In the Term group, estradiol (r_s_ = −.415, *p* = 0.028) and progesterone levels (r_s_ = −.396, *p* = 0.037) were significantly inversely correlated with BDI scores. In the Preterm group, prolactin levels were significantly negatively correlated with scores of BDI (r_s_ = −.535, *p* = 0.022) and STAI-S (r_s_ = −0.494, *p* = 0.037) shown in **Figure 4**.

### Group Comparisons Between Fathers

#### Depression, Anxiety, and Stress Scores in Fathers

**Figure 5:**
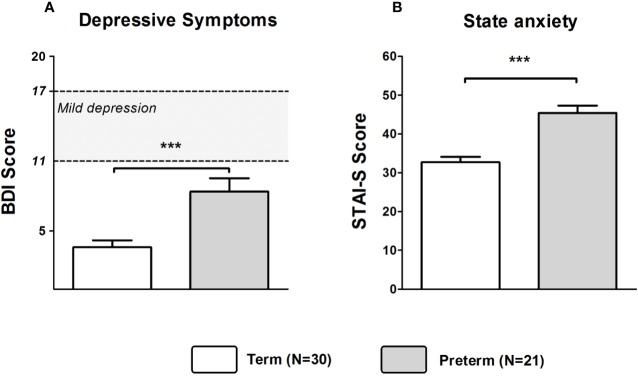
Scores of BDI **(A)** and STAI-S **(B)** in fathers of infants born at term or preterm. ***p < 0.001. Data are shown as mean values ± SEM.

Mann–Whitney U tests revealed that depressive symptoms were more pronounced in fathers of the Preterm group compared to the Term group (Z = −3.375, *p* < 0.001). However, BDI scores were in both groups well below the cutoff for mild to moderate depression (see **Figure 5A**). Furthermore, STAI-S scores (Z = −4.301, *p* < 0.001) were significantly higher in fathers of the Preterm group compared to the Term group, indicating higher levels of anxiety in new fathers of preterm infants (**Figure 5B**). Fathers in the Preterm group also showed significantly elevated levels of perceived stress when compared to the Term group (Z = −2.912, *p* = 0.004).

#### Post-Awakening Cortisol Secretion in Fathers

Groups of Preterm and Term fathers did not significantly differ in the CAR (all *p* > 0.05).

## Discussion

The main purpose of the present study was to provide novel insights into potential differences in depression, anxiety, and perceived stress as well as endocrine markers between new parents of very preterm and term born infants. As expected, mothers in the Preterm group experienced higher psychological distress than mothers of term infants. The mean value for depression even reached a clinical level, indicating mild depression in 55% of mothers in the Preterm group. For the STAI-S, even 72% of mothers in the Preterm group exceeded the cutoff of 45. Additionally, levels of perceived stress were significantly higher in mothers and fathers of preterm infants. In line with previous results we found that premature birth and associated adverse effects on both parents and child account for more pronounced psychological distress in preterm mothers (25). In particular depression but also anxiety in mothers are assumed to cause various consequences e.g., disturbances in attachment of mother and their babies and thus putting the long-term development of their babies at risk (38, 39).

In mothers of infants born at term, scores of depression correlated negatively with levels of estradiol and progesterone. This is in accordance with results from previous studies proving that a withdrawal of estradiol and progesterone may trigger depressive states (7). Furthermore, a treatment with estradiol improved symptoms of PPD (8, 9).

Surprisingly, in mothers of preterm infants this relationship could not be established. The significantly lower concentrations of estradiol and progesterone in mothers of preterm babies might merely be attributable to birth in an earlier gestational week and scores of depression may go back to higher psychological distress that mothers in the Preterm group are exposed to. Due to the small sample size, these results should be interpreted with caution.

However, in our study prolactin correlated negatively with depression and anxiety in mothers of preterm infants. Prolactin has anxiolytic and stress-relieving properties (2). A previous study reported a negative correlation between anxiety and prolactin in women giving birth at full term (11). For PPD only few studies with inconsistent results exist (12). Yet, two previous studies found that mothers of term infants with high depression scores showed lower levels of prolactin four to six weeks after birth (40, 41). Unfortunately, the aforementioned studies did not control for breastfeeding and prolactin was not measured in the immediate days after birth. Thus, one may speculate that non-breast-feeding mothers might exhibit even more severe symptoms of depression or anxiety.

For TSH, no significant differences between preterm and term mothers were found. However, this is not surprising since, in an earlier study, associations between TSH levels shortly after delivery and symptoms of depression 6 months after delivery were only found in women with levels outside reference ranges over 4.0 mU/L (15). In the study herein, we have excluded women who were treated pharmacologically because of hypo- or hyperthyroidism during pregnancy. Thus, mothers with exceptionally high or low levels of TSH were not present in our sample. Additionally, further analyses at later time-points could lead to a detection of a possible relationship between mood disturbances and initial levels of TSH postpartum. It has to be noted that levels of estradiol, progesterone, prolactin and TSH rise during pregnancy and return to baseline levels after birth (7, 42–45). Due to these dynamic changes of the endocrine system throughout pregnancy and puerperium difficulties arise when trying to integrate biological measures and psychological consequences of having a child (46, 47).

At awakening, the salivary cortisol concentrations did not differ between groups of mothers. However, the CAR in the first hour after awakening was significantly higher in the Preterm than in the Term group. Unfortunately, despite extensive research until now it was not possible to establish a unique pattern of the CAR after parturition for women known to experience psychological distress or even suffering from mental disorders (27). Contrasting our results, in mothers giving preterm birth a considerable dysregulation of the hypothalamic–pituitary–adrenal axis (HPA-axis) could be detected, leading to a significantly diminished AUCg for salivary cortisol on day six after delivery compared to mothers giving term birth (48). These differences might also go back to non-compliance in saliva sampling. With several disturbances occurring generally in women after delivery, which are augmented by the stressful experience of being fearful and uncertain about their babies’ health in preterm mothers, compliance most likely decreases and could lead to misaligned cortisol values (49). However, a delay of saliva sampling in our study could not be found. Based on self-report questionnaires, all parents closely adhered to the time schedule of the saliva collection protocol.

Even though mothers in the Preterm group markedly differed from mothers in the Term group regarding estradiol, progesterone, prolactin and the AUCg and AUCi, we only found significant associations between prolactin levels and depression as well as anxiety in mothers of preterm infants. Since we could not establish a causal relationship, we cannot be sure that there is an immanent connection of neuroendocrinological and psychological measures.

To our knowledge, only few studies have assessed psychological and physiological parameters in new fathers (17, 25). Nevertheless, a number of risk factors have been identified that may contribute to the development of PPD in fathers, including increased demands regarding household chores and worries regarding health issues of mother and child (50, 51). Additionally, depression in mothers seems to be the strongest predictor for paternal depression (52). Compared to fathers of term infants, fathers of preterm babies reached higher levels of depression, anxiety and stress. While mean scores of depression were below clinical relevance, anxiety was above the cutoff in the STAI-S affecting about 53% of fathers in the Preterm group. However, in contrast to mothers, we could not find evidence for an altered cortisol secretion in fathers of preterm infants.

### Limitations

The study has notable strengths such as the inclusion of fathers and the assessment of endocrine parameters in addition to psychological measures. Nevertheless, there are also some limitations that should be mentioned. First, due to a possible selection bias and moderate sample sizes, a generalization of our results should be done cautiously. Since participation in our study required additional effort, those parents who experienced higher psychological distress might have had a stronger tendency to drop out of the study or did not even participate at all. However, this is a common problem and high dropout rates have been reported earlier for alike populations (21). A further shortcoming of our study is a lack of assessments prior to birth. This relates to physiological measures and the psychological status, which might lead to preterm birth. However, biomarkers only have a limited capacity to predict preterm birth (29, 30, 53). Nevertheless, by gaining information from self-reports we were able to exclude participants with known psychiatric or mental disorders. However, given the higher reliability and validity in the assessment of psychiatric lifetime disorders, clinical interviews should be used for the assessment of mental health in future studies (25). Furthermore, in our study we did not exclude individuals with a body mass index (BMI) above 25. Even though groups did not differ significantly regarding the BMI, obesity in mothers might be associated with the CAR. Future studies should use prospective designs to examine potential associations. Obviously, the data presented in this paper are cross-sectional, focusing only on the early phase immediately after birth. It remains unclear whether our results can be attributed to exclusively physiological, psychological, or intermingled features of both. Taken together, it remains unclear whether there were preexisting vulnerabilities or symptoms of anxiety and depression are the consequence of having a preterm infant. Regardless of possible associations prior to birth our results show that women who gave birth preterm show elevated levels of depression, anxiety and stress. Longitudinal follow-up studies throughout pregnancy need to assess the role of endocrine markers and whether the observed psychological disturbances also impact on long-term mental health of preterm mothers and fathers.

## Conclusions

Taken together, our findings provide novel evidence that new mothers and, importantly, also new fathers of preterm infants suffer from a higher burden of psychological distress than parents of full-term infants. Depressive, anxiety, and stress symptoms in mothers of preterm infants might result from worries about the health of their child, limited skin contact or being dependent on clinical staff, but might be also linked to altered endocrine profiles such as lower serum levels of estradiol, progesterone, prolactin, and TSH as well as a heightened CAR.

Regardless of the causation of postpartum depressive, anxiety, and stress symptoms, mothers and fathers of preterm infants should receive adequate intervention including a close monitoring of endocrine and psychological measures. Further studies are needed to show if interventions during the first weeks after birth are able to change the trajectories of posterior symptoms of depression and anxiety. Hopefully, this will lead to a better understanding of the psychophysiological underpinnings of affective disturbances in parents of babies born preterm and will help to ameliorate psychological distress in the postpartum period.

## Data Availability Statement

The datasets generated for this study are available on request to the corresponding author.

## Ethics Statement

The study protocol has been approved by the Ethics Review Board of the University of Duisburg-Essen (approval no. 09-4239) and was performed in accordance with the Declaration of Helsinki. All subjects gave written informed consent. The protocol was approved by the Ethics Review Board of the University of Duisburg-Essen.

## Author Contributions

UF-M, MS, and HE designed the study. TW provided the statistical analysis and interpretation of data, and wrote the manuscript. TW, NS, and AS executed and supervised the acquisition of data. TW, NS, AS, UF-M, MS, and HE contributed to the conception and design of the study, interpretation of data, and internal revision of the manuscript. All authors contributed to and have approved the final manuscript.

## Funding

The work was partly supported by an award from the German Association for Psychiatry, Psychotherapy and Psychosomatics (DGPPN). The funding organization was not involved in study design; in collection, analysis, and interpretation of data; in the writing of the report; or in the decision to submit the article for publication.

## Conflict of Interest

The authors declare that the research was conducted in the absence of any commercial or financial relationships that could be construed as a potential conflict of interest.
